# Beyond patient-sharing: Comparing physician- and patient-induced networks

**DOI:** 10.1007/s10729-022-09595-3

**Published:** 2022-06-01

**Authors:** Eva Kesternich, Olaf Rank

**Affiliations:** Institute of Economics and Business Administration, Albert-Ludwigs-University of Freiburg, Rempartstr. 16, 79098 Freiburg, Germany

**Keywords:** Patient- and physician-induced networks, Outpatient sector, Service complementarity, Closed and open triads, Exponential random graph models

## Abstract

The sharing of patients reflects collaborative relationships between various healthcare providers. Patient-sharing in the outpatient sector is influenced by both physicians’ activities and patients’ preferences. Consequently, a patient-sharing network arises from two distinct mechanisms: the initiative of the physicians on the one hand, and that of the patients on the other. We draw upon medical claims data to study the structure of one patient-sharing network by differentiating between these two mechanisms. Owing to the institutional requirements of certain healthcare systems rather following the Bismarck model, we explore different triadic patterns between general practitioners and medical specialists by applying exponential random graph models. Our findings imply deviation from institutional expectations and reveal structural realities visible in both networks.

**Supplementary Information:**

The online version contains supplementary material available at 10.1007/s10729-022-09595-3.

## Highlights

- Our study focuses on one patient-sharing network and distinguishes between two mechanisms causing the formation of this network: physicians’ and patients’ activities.
- Our study contributes to research examining whether institutional expectations are fulfilled in networks arranged either by physicians or patients by considering various exchange possibilities among general practitioners and medical specialists in treating common patients.
- By using a network analytical approach and going beyond the dyadic level enables us to detect how office-based physicians cluster when the principle of service complementarity is included in triadic exchange.
- Our findings suggest that patients tend to decide for themselves which physicians to consult and referral and thus coordination by physicians seems to be the exception. Patients do not necessarily follow the principle of service complementarity but connect physicians that occupy the same institutional role.
- The exploration of the patient-induced network reveals “hidden” treatment paths not perceived by physicians. This could be used to locate existing network patterns and draw physicians’ attention to them.

## Introduction

Multiple actors are integrated in the provision of medical care. Healthcare providers typically work together sharing their resources and knowledge to ensure comprehensive healthcare [1]. One type of interaction that has increasingly being studied as part of investigating networks between healthcare providers is patient-sharing [e.g. 2]. A patient-sharing tie occurs if two healthcare providers treat the same patients [3]. The sharing of patients involves the exchange of (interdisciplinary) medical expertise and information. Communication, mutual consultation, and coordination are required to organize healthcare delivery [4]. This kind of exchange is classified as “a reliable signal of collaboration” between healthcare providers [5]. Though prior studies have examined (non-medical) determinants [e.g. 6] and consequences [e.g. 7] of patient-sharing networks, it is still unclear how the patient impacts the formation of these relationships. In healthcare systems in which patients enjoy a high degree of autonomy, e.g., the ability to select their own physician, the patient’s activity and behavior also determines the structure of a patient-sharing network. Especially in systems following the Bismarckian tradition, the free choice of one’s physician is a central element [8]. Although optional gatekeeping arrangements are increasingly being implemented, the patient’s freedom of choice often remains intact [9].

We have taken this as a reason to investigate the structure of a patient-sharing network by differentiating between a physician- and patient-induced network. We study one patient-sharing network and focus on two different mechanisms that allow us to examine the network structure in greater detail: One mechanism reflects the activities and behavior of physicians. In such cases, we use the term “physician-induced network”. A second mechanism represents the activities of patients. In this context, we focus on a network that is based solely on the actions of patients, defined as a “patient-induced network”.

Based on the principle of service complementarity, general practitioners (GPs) and specialists commonly work together in small groups. Since physicians interact with numerous other physicians and are differently embedded in the network, we study patient-sharing beyond the dyadic level [10]. The triadic perspective broadens the dyadic view by focusing on three (complementary) actors that may be connected [11]. We explore the extent to which certain triadic tendencies appear in both networks. This serves as a basis for assessing whether physicians and patients adhere to certain institutional guidelines and expectations rooted in general health policies.

In order to compare the triadic structures in physician- and patient-induced networks, we derive a regional patient-sharing network among office-based physicians in Germany by using medical claims data. For our empirical analysis, we apply a class of exponential random graph models (ERGMs) that is particularly appropriate for investigating the structure of social networks [12]. The results partly confirm that the outpatient course of treatment corresponds to the institutional requirements imposed on physicians and patients. However, some results are inconsistent with the guidelines, indicating that other factors may affect the triadic exchange among office-based physicians. We discuss these “structural realities” and formulate alternative scenarios that might be responsible for the deviations.

## Dimensions and structures of patient-sharing

### The complexity of patient-sharing relations

The combined treatment of a patient by multiple physicians leads to linkages between these physicians. When the patient receives treatment from both physician A and physician B, these physicians are connected [3]. Patient-sharing networks represent exchange practices involving physicians and serve as a basis for investigating patterns of interaction [e.g. 2]. With patient-sharing as a routine process in itself, physicians can exchange information and experiences, and provide mutual advice. They communicate with each other and disseminate knowledge. Thus, this type of relation serves as an indication of collaboration, encompassing different forms of interaction [2, 13].

While a variety of empirical studies have focused on patient-sharing in hospital settings [14], we examine patient-sharing patterns in the ambulatory environment. Considering transfer relations in terms of inpatients moving within and between hospitals, medical professionals initiate further treatments in the majority of cases, particularly across organizational boundaries [5]. The decision to refer a patient is usually a choice made by both the sender and receiver hospital. Lack of available resources and capacities, such as adequate medical knowledge or qualified professionals, can often necessitate follow-up treatments at partner hospitals. Transfers are mainly regulated and coordinated by the hospitals [5].

In the outpatient sector, patient-sharing relations represent multidimensional exchange between office-based physicians. The occurrence of this type of relation can be a result of activities by both physicians and patients. Both parties can bear responsibility for the establishment of the network [15]. The impact of the patient side in outpatient healthcare can play a decisive role in identifying and explaining possible interactions.

Each healthcare system adheres to certain institutional regulations and laws, thereby significantly shaping behavior and interactions between actors. These “institutional idiosyncrasies” [5] have to be considered in any detailed understanding of networking mechanisms in outpatient care, since these also determine actors’ behavior and consequently the structure of the network. Regarding countries following the Bismarck model, patients are often entitled to freely choose a physician for treatment. The patient's free choice of physician can be applied to both primary and specialist care, for instance in Germany [16]. This is a fundamental principle allowing patients to actively influence treatment pathways. Such freedom is restricted by institutional regulations in many other healthcare systems, often rooted in the Beveridge model [17]. An example of this is the gatekeeping-principle, which requires the patient to make first contact with the assigned GP [18]. Research into patient-sharing in healthcare systems following the principles of free choice implies that both the physician and patient sides can be addressed. The sharing activity has to be considered from both the physician's and the patient's perspective.

### Physician- and patient-induced networks

Physician-induced relationships are formed first and foremost due to medical reasons such as the need for specific diagnostics and therapeutic measures [14]. Insufficient medical know-how, diagnostic uncertainty, and resource bottlenecks cause physicians to consult fellow physicians for advice on common patients [19]. To ensure complete, efficient, and high-quality treatment, physicians frequently advise physicians from other disciplines. They pool (interdisciplinary) knowledge, deliberate, and make joint decisions to guarantee continuity of care [2, 7]. Physician-induced interrelations come about frequently through an arranged referral to another physician, who possesses a specialization necessary to (further) treat the patient [20]. It is usually the physician who instigates follow-up treatment by giving the patient a referral. In some cases the patient might request that the physician provide a referral, it is mainly the physician who is responsible for enlisting the services of a second physician [21]. The physician contacts his or her peers using various communication channels (doctor’s letter, telephone, email) and coordinates the progress of treatment. In addition to medical reasons, organizational and financial factors such as capacity and budget constraints also influence the shared treatment of patients, stemming predominantly from the medical side [22]. For this reason, we define physician-induced patient-sharing as network ties established in the first instance by the physician’s actions and behavior.

Patient-induced networks can be traced back to the actions of patients and should be classified in a different manner. Due to specific institutional arrangements, patients’ behavior can affect the treatment process and consequently the structure of the relationships between outpatient providers. In many healthcare systems, the individual patient enjoys a high degree of decision-making autonomy being able to select the physician best suited to his or her needs even without a referral [23]. An exception is, for example, if the patient participates in a planned and formalized treatment program and interdisciplinary care is predetermined by certain physicians [24]. Even though structured treatment models have increasingly found their way into various healthcare systems in recent years and are supported by health system administrators and policymakers through corresponding reforms and initiatives, in many cases patients themselves still decide which physician to consult [25]. Although medical ambiguities and variables often explain why patients visit multiple physicians, several other factors contribute to the understanding of patient-sharing as a cooperative construct between physicians. Personal preferences and expectations impact the choice of physician and utilization of healthcare services [26]. However, uncertainty regarding confirmation of the given medical diagnosis might lead the patient to consult further specialists in addition to the GP’s examination, though this might not seem necessary to provide the patient with comprehensive care. Thus, obtaining second opinions can lead to over- and misuse of outpatient services. The influence of the social environment and digital media is another factor that encourages patients to define their own treatment options. Patients are prone to follow recommendations and (online-) evaluations when consulting a physician [27]. Patients’ experiences, attitudes, and behavior are therefore crucial when it comes to understanding network structures among physicians based on shared patients.

### Service complementarity and triadic closure or open paths

We examine the structural regularities of a patient-sharing network by differentiating between the physician- and patient-induced network structure. The identification of certain micro configurations leads to an understanding of the structural logic of physician- and patient-induced networks [28]. Office-based physicians are required to collaborate for delivering comprehensive care to patients. They are involved in treating a broad spectrum of diseases, necessitating exchanges with physicians of diverse medical disciplines [29]. These interactions result in group formations, clusters, and sub-networks between physicians [11].

The basis for different group-level structures within networks is the dyadic level [30]. The sharing of patients implies that at least two physicians are connected to each other, indicating a dyadic relationship. The pairwise interaction often occurs between providers with differing specializations and medical knowledge [10]. The exchange of complementary resources constitutes a strong driver for the creation of patient-sharing ties [31]. The principle of service complementarity becomes apparent when GPs and medical specialists share patients and hence their capabilities, knowledge, and resources. With respect to the dyadic level, we assume that sharing patients primarily takes place among complementary physicians, either between GPs and specialists or between specialists of dissimilar medical disciplines. The former interaction type results from the central function of the GP in outpatient care. They are often the first point of contact for patients and ideally take over the coordination of the following course of treatment [32]. The second case occurs when patients consult a second specialist after they have already been treated by another specialist, a step that may seem necessary to complete the treatment. It may also be the case that two specialists share their knowledge and communicate with each other owing to different medical expertise required for continuing patient treatment [33].

Considering service complementarity in a network among office-based physicians, in many cases, more than two physicians are involved in the treatment process. One reason for this is that complex or chronic diseases often demand interdisciplinary outpatient care [34]. Furthermore, GPs are connected to different specialists, as they contact their peers after an initial diagnosis, give a referral, or have patients who take it upon themselves to visit a specialist, thus coupling those physicians. In this way, GPs can indirectly link various specialists with each other and function as gatekeepers [18]. Since GPs and specialists interact with numerous physicians and are differently embedded in the network, we investigate patient-sharing beyond the dyadic level [10]. The triadic level extends the dyadic perspective and incorporates three actors who are possibly linked. Dyadic connections are part of triadic structures [35]. The triad represents an important microstructure for analyzing networks because it can be assigned between pairs of actors and higher-order network patterns [36].

A triad consists of three actors. All three actors can be tied to each other, which leads to a closed triad. If one pair of actors is not connected, the result is an open triad. This triad type reflects the theory of structural holes, stating that one actor binds two unconnected actors closing structural holes within a network. This represents the role of a broker or gatekeeper [37]. A third option is that only two actors are linked and one actor is isolated from the dyadic pair. It is also possible that all three actors are not tied to each other. Accordingly, four different triad types, known as the triad census, can be present in undirected networks [11].

We focus on open and closed triad formations. In addition, we integrate information concerning whether the outpatient providers are GPs or specialists. In other words, we combine the selected triad types with the institutional role of office-based physicians [38]. We follow this approach for various reasons: First, we explore the extent to which open and closed triads coexist in undirected physician- and patient-induced networks in order to improve our understanding of extra-dyadic interactions among physicians providing outpatient care. Second, we demonstrate the role of complementary exchange between physicians in both networks by examining open and closed triadic patterns. Moving beyond the dyadic level allows us to draw more specific conclusions about the sharing behavior of physicians, as they are tied to multiple other physicians, thus forming subgroups within the network. Third, by considering closure and open-paths mechanisms between GPs and medical specialists more precisely, we highlight the extent to which institutional expectations are fulfilled.

The institutional framework of a healthcare system contains specific conditions that can determine physicians’ and patients’ behavior [39]. With regard to outpatient care provision, physicians generally have to follow specific rules to ensure efficient, cost-effective, comprehensive, and high-quality care [40]. These guidelines are often established by multiple actors involved in structuring and managing outpatient healthcare, such as health insurance agencies, policymakers, public authorities and, physicians’ and patients’ representatives. This depends on which model of regulation is adopted to organize healthcare [41]. Although all these stakeholders pursue their own interests, there are some universal institutional views on how the utilization and delivery of healthcare should proceed. In general, access to healthcare should be universally guaranteed for all patients [42]. This task is performed in many healthcare systems by GPs and specialists working in different practice settings. Over- and misuse should be avoided in order to reduce waiting times and provide resources for patients who require specific treatment [43]. Some countries have introduced gatekeeping systems, which manage care processes and are often regulated by contract. If a patient voluntarily participates or is compelled to take part in such a program, the GP is the first point of contact. The specialist can be consulted subsequently [17]. In many countries, the choice of physician is partly or completely restricted. In other systems, the patient is entitled to freely select a GP and a specialist and can decide which physician he or she wishes to consult [41]. If the choice of a physician is not at all or only marginally restricted, the patient might influence the relationship between complementary physicians. The interaction patterns among GPs and specialists can therefore differ, depending on whether the exchange processes are initiated by physicians or patients.

Differences can be identified by examining patient-sharing beyond the dyadic level. A focus on triadic mechanisms is a possible means of investigating physician- and patient-induced networks in more depth. This is for two main reasons: First, we expect no significant differences between physician- and patient-induced networks if we solely consider dyadic exchange among GPs and specialists in healthcare systems in which patients can freely decide where to go for treatment. We assume that dyadic complementary exchange is present in both networks. Physicians interact with numerous physicians, especially across disciplines, and patients frequently visit a GP and a specialist in order to obtain all the necessary treatment [44]. However, the investigation of pairwise exchange reveals no indication of which additional physicians are contacted or to what extent further physicians are involved in the treatment process. To gain more insight into the further course of treatment, our study focuses on open and closed triadic patient-sharing patterns.

Second, the study of these substructures enables the identification of possible deviations from institutional expectations. Regarding physician-induced networks, we do not expect three GPs to group and share patients, thus building a closed triad. The exchange rather pursues the principle of service complementarity in networks initiated by office-based physicians. It is more likely that three providers with differing specializations will share patients with each other, for instance, due to a need for different varieties of medical expertise [2]. We also assume that GPs often take over the role of gatekeepers, regardless of whether the patient participates in a structured treatment program or not, thus involving them in a triadic interaction. Considering the patient-induced network structure, we do not anticipate that three GPs will be linked by common patients. Patients are advised to avoid consulting physicians within the same discipline to obtain a second opinion, for example. Moreover, complementary exchange should be ideally initiated by the physician, for instance in the form of a referral [45]. This means that patient-induced triadic relationships between different GPs and specialists should ideally be avoided. However, due to the free choice of physician, it is possible, that the patient autonomously visit specialists and thus closed triads between a GP and different specialists can occur in a patient-induced network [17].

The same applies to open triad formations in patient-induced networks. These chain-like linkages between three actors can be composed of different physicians. Multiple examinations by the same provider type should be prevented, for instance, to save expenses and avoid capacity utilization [41]. For this reason, GPs are not intended to share patients with other GPs who are not linked if the relationship is caused by the patient’s activities. The same applies to open triads with specialists included in patient-induced networks. The treatment pathway of a patient should ideally be determined and coordinated by the physician [46, 47]. But based on the free choice of physician, patients choose their specialists themselves without having a referral from their GP. There may be cases in which patients visit two specialists of the same discipline to obtain second opinions or because they were dissatisfied with the first specialist treatment [17]. The occurrence of an open triad, in which a GP links two specialists, is therefore also possible in patient-induced networks.

In addition, the gatekeeper behavior of GPs can also be observed in physician-induced networks [48]. However, we do not expect that a treatment pathway will consist of only three GPs. Based on institutional expectations towards physicians and patients, diverse closed and open triad configurations should be present or absent between primary and specialist care.

## Empirical setting, data, and methods

### Data source and research setting

Our empirical analysis draws on medical claims data from 2016 provided by the National Association of Statutory Health Insurance Physicians. The administrative data comprises patient- as well as provider-related information for about 70 million patients covered by statutory health insurance in Germany [49]. Since health insurance is mandatory in the German healthcare system, the available data allows a complete overview of the statutorily insured population [50]. The database permits one to undertake a complete investigation of all relationships between a specified number of physicians, required for the study of networks using specific analytical methods [28]. To compare physician- and patient-induced networks, we focused on a regional patient-sharing network within a rural district in Germany.^1^ In the German outpatient system, rural areas are particularly affected by supply bottlenecks and gaps in healthcare provision [51]. Thus, the investigation of networks in rural environments is especially valuable for identifying any deviations from institutional expectations and to derive appropriate interventions to improve the regional healthcare infrastructure. We selected a district region as these are used for the planning and administration of outpatient care provision and can therefore be applied to define the network boundary [52]. Previous studies which have focused on patient-sharing networks mainly in hospital settings, already have chosen institutionally defined administrative units or regions to determine the network [e.g. 22]. In addition, routine outpatient care is usually provided at the local level and across organizational boundaries [41]. Accordingly, the selection of a district represents a regular case for analyzing the structure of a patient-sharing network in the outpatient healthcare sector. Furthermore, the German system “represents the most typical Bismarckian case because of its historical origins and the greater autonomy of the sickness funds” [53]. Countries that have implemented such a health insurance system allow patients to choose their GP and specialist freely or to restrict their free choice only marginally [17, 41]. Consequently, the selected region forms an ideal foundation for creating patient-sharing networks induced either by physicians or by patients.

### Network data

**Fig. 1:**
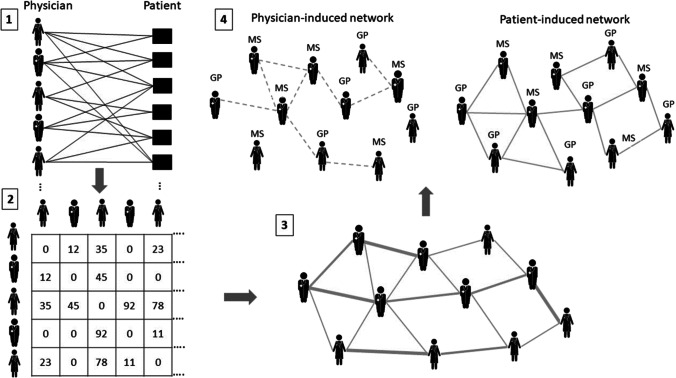
Schematic representation of patient-sharing network creation and differentiation. *Notes*: *Step 1*: From the patient-physician bipartite network, we first generated a network linking physicians based on common patients (unipartite network). *Step 2/3 (table and illustration)*: The result is a weighted network among physicians. *Step 4*: To form a patient- and physician-induced network, we utilized the referral information available in the accounting data. This means that patient-sharing links that came about due to referrals are classified rather as physician-induced ties. Patient-sharing ties without any referral details can be assigned to patients’ choices. GP = general practitioner; *Step 4*: MS = medical specialist;  = with referral;  = without referral; Own development, based on Moen et al. [3]; Casalino et al. [55]

In line with previous research [e.g. 54], we deduced a patient-sharing network among office-based physicians from medical claims data. We selected children and adolescents as the patient population in this study to delineate the network. Many previous studies that have empirically analyzed patient-sharing networks have addressed particular diseases or diagnoses to determine network actors [e.g. 3, 26]. Consequently, certain physicians participating in outpatient healthcare delivery might be ignored. We aim to create a network of physicians who are primarily responsible for regular outpatient care. The focus on pediatrics ensures that a broad spectrum of specialists is considered, and that not only certain specialists are included in the network. We integrated these providers by means of the selected patient population. A few specialists have been removed from the network since they do not directly interact with patients or because their availability is determined by other planning levels [29]. We have also excluded treatment cases not reflecting the routine treatment processes, such as laboratory services or emergency cases. As a result, 113 physicians with differing specializations are included in the patient-sharing network, overseeing the provision of ambulatory healthcare for children and adolescents. For the creation of the network, we draw upon studies by Barnett et al. [33], Casalino et al. [55] and Moen et al. [3]. Figure 1 depicts a schematic representation of how we have constructed the networks. From the patient-physician interactions (bipartite network), we first generated a network linking physicians based on common patients (unipartite network) [44]. The result is a weighted network among physicians since the majority of physicians usually share more than one patient within a year. To form a patient- and physician-induced network, we utilized the referral information available in the accounting data. This means that patient-sharing links that came about due to referrals are classified rather as physician-induced ties. The referral documentation is representative of direct interaction and communication (e.g. through a referral form or physician’s letter) between physicians [33, 48], whereas patient-sharing ties without any referral details can be assigned to patients’ choices. Due to the fact that no sender and receiver information was provided, for instance, through temporal details of the transfers, we obtain an undirected network between individual physicians. We dichotomized the network because we are not interested in the intensity or frequency of the exchange, but rather whether a tie is present or absent between a pair of physicians. A relationship is present if two physicians have shared at least one patient in 2016 [38, 56]. The application of other dichotomization criteria, such as the usage of a particular threshold value to determine a relationship, can result in an exclusion of important actually occurring partnerships in the network [57]. Network data was arranged in a binary adjacency matrix of order 113 × 113. Each cell in the matrix corresponds to a relation between two physicians *i* and *j* (*x*_*ij*_). If *i* and *j* have commonly treated at least one patient, cell *x*_*ij*_ was coded as 1; otherwise, *x*_*ij*_ was coded as 0.

### Variables and measures

#### Service complementarity

To investigate closed and open triad formations among complementary physicians, we classified the physicians as GPs or medical specialists based on their institutional role [38]. Since we have chosen children and adolescents as the patient population, we designated pediatricians as GPs. Pediatricians often serve as first contact for this group of patients and provide primary care as well [1]. All other physicians who provide predominantly specialist care belong to the second category. We formed a binary variable with the value 1 for GPs and 0 for medical specialists. Regarding service complementarity, physicians who can be assigned to distinct levels of care share patients with each other. In the case of outpatient care, exchange among GPs and specialists is to be expected [29].

#### Service complementarity and closed or open triads

When incorporating the binary attribute into triadic substructures possibly occurring in patient-sharing networks, different types of closed and open triadic configurations can emerge [58, 59]. Based on general institutional expectations, we assume that some triadic structures are more likely to be found in physician- or patient-induced networks than others. To explore the possibilities of how GPs and specialists interact when the focus shifts to triadic patterns, we identified three closed and three open triad forms. In particular, we included a closed triad involving only GPs to capture the tendency of GPs to form a clique or a small group (*GGG-triad*). If physicians and patients adhere to the general regulations, this triad configuration should not be observed. Additionally, we integrated a closed triad consisting of two GPs and one specialist (*GGS-triad*). We expect this configuration to be present in physician-induced rather than in patient-induced networks. Due to service complementarity and knowledge gaps, two GPs and one specialist group together through referral transfers. Referrals between GPs should only occur in cases where GPs provide special treatment and the patient receives appropriate treatment from another GP [45]. In contrast, multiple examinations initiated by the patient should be avoided [41]. The same is valid for the presence of a closed triad between one GP and two specialists (*GSS-triad*). Concerning the physician-induced network, the GP is supposed to act as gatekeeper and connect different, potentially complementary specialists with each other [60]. Ideally, the patient should seek specialist advice following a referral, but in consideration of patients’ right to choose their own physician, this closed triadic structure is likely to also be present in patient-induced networks.

Regarding the open triad structures, we predict similar outcomes. We included an open triad covering the propensity of GPs to form chain-like structures exclusively among themselves (*GGG-chain*). The occurrence of this effect is rather unlikely in both networks. By contrast, open triads involving GP-specialist interactions can be observed when patient treatment is initiated by the physician. In detail, we consider an open triad type in which a GP is the link between another GP and a specialist, hence indirectly connecting them (*GGS-chain*). Furthermore, we integrated a third open triad effect in which a GP links two specialists, possibly acting as a gatekeeper (*SGS-chain*). Owing to institutional requirements, the last two open triadic structures tend to occur in patient-sharing networks determined by the physician. Regarding the patient-induced network, multiple examinations by the same provider should ideally be avoided and patients normally do not consult two GPs. The presence of the *GGS-chain* effect is therefore unlikely in this network. But patients autonomously visit different specialists following treatment by a GP. Based on this, the *SGS-chain* effect is likely to occur in the patient-induced network. Table 1 lists the various expectations for the physician- and patient-induced network.

**Table 1:**
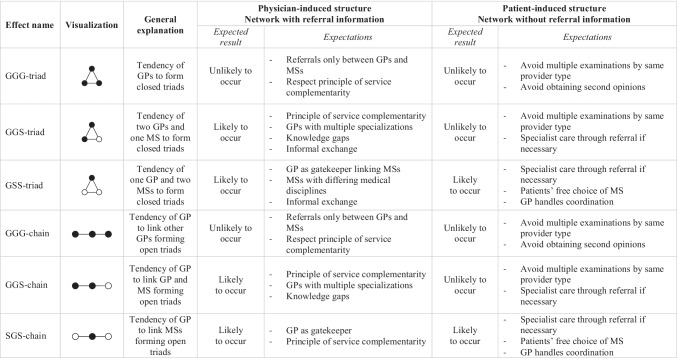
Overview of triadic structures for physician- and patient-induced network and expected results

● = general practitioner with pediatricians (G/GP); ○ = medical specialist (S/MS)

#### Further actor-related attributes

Although we expect the interplay between service complementarity and various triadic patterns to play a key role in understanding the physician- and patient-induced network structure, further actor-related characteristics may affect the formation of patient-sharing ties.

In addition to licensed and authorized physicians, employed physicians are increasingly providing outpatient care [61]. We have inserted a categorical variable differentiating between the possible status forms arising in the German outpatient care sector to capture the tendency of physicians with the same status to share patients.

Moreover, we added the organizational form of the medical practice where the individual physician works as categorical variable. We did so to capture the tendency to share patients among physicians providing services in practices with different organizational forms. The shared treatment often occurs between single and group practices or ambulatory healthcare centers [62]. The different practice forms are based on the official classification of the National Association of Statutory Health Insurance Physicians.

In addition to the complementary exchange between GPs and specialists, we also integrated the medical specialty of each physician as a categorical variable in order to test whether physicians with dissimilar specialties are more likely to be related through common patients.

Several scholars have already shown that spatial proximity influences the formation of relationships among healthcare providers [e.g. 22, 32]. We argue that local reachability and direct access to outpatient healthcare are crucial for both patients and physicians. Geographical distance was measured using travel times in minutes [14, 63]. We arranged the distances between each pair of physicians in a weighted adjacency matrix and included this variable as a dyadic covariate.

### Empirical analysis: Exponential random graph models

We applied a class of ERGMs to explore the importance of open and closed triadic substructures in physician- and patient-induced networks. The application of ERGMs is widely used, for instance, in organization and management studies [e.g. 64]. It is also being increasingly utilized in studies examining network formations in healthcare settings [e.g. 65].

Using ERGMs is particularly appropriate for analyzing the regular structural patterns arising within a network, as this statistical method accounts for tie interdependence in network structures. In comparison to common statistical methods such as regression analysis, observations of network relations are not independent from each other [12, 28]. We also made use of this approach to highlight the extent to which the various triadic patterns characterize the overall network structure. Applying ERGMs enables one to simultaneously test the impact of actor-based attributes and structural patterns on tie formation. This means that network effects are tested in dependence of other effects using one statistical model [58]. In detail, this method allows for the identification of local subgraphs represented by specific parameters in the model that explain the structural logic of a network. Therefore, a positive (negative) parameter estimate shows that the selected configuration occurs more often (less often) than expected by chance. ERGMs reflect the probability of an overall network structure through parameters attributed to certain substructures of the network. Based on the given observed network structure, modeling ERGMs is a stochastic process in which the presence of a relational tie is influenced by the presence or absence of other network ties and actor-based attributes: “Using these models, one can infer whether a configuration occurs in the network more than expected by chance, given the other effects in the model” [66].

In addition to the open and closed triadic substructures, we included further actor-related (exogenous) and structural (endogenous) effects that possibly have an impact on the formation of physician- and patient-induced networks. Regarding the actor-related characteristics, we integrated configurations representing homophily (matching effect) or heterophily (mismatch effect) and, additionally, an activity effect, which captures the network activity of an actor who exhibits a specific attribute. A dyadic covariate was included for examining the influence of distance on the creation of patient-sharing ties in both networks. Furthermore, we inserted three structural effects in our models. The *two-path* and *alternating two-paths* effects capture the tendency of physicians to share patients through (multiple) other physicians but not directly. To account for variation in the degree to which a physician is linked to others, we integrated an *alternating stars* effect. Table 2 gives an overview of the effects included in our models.

**Table 2:**
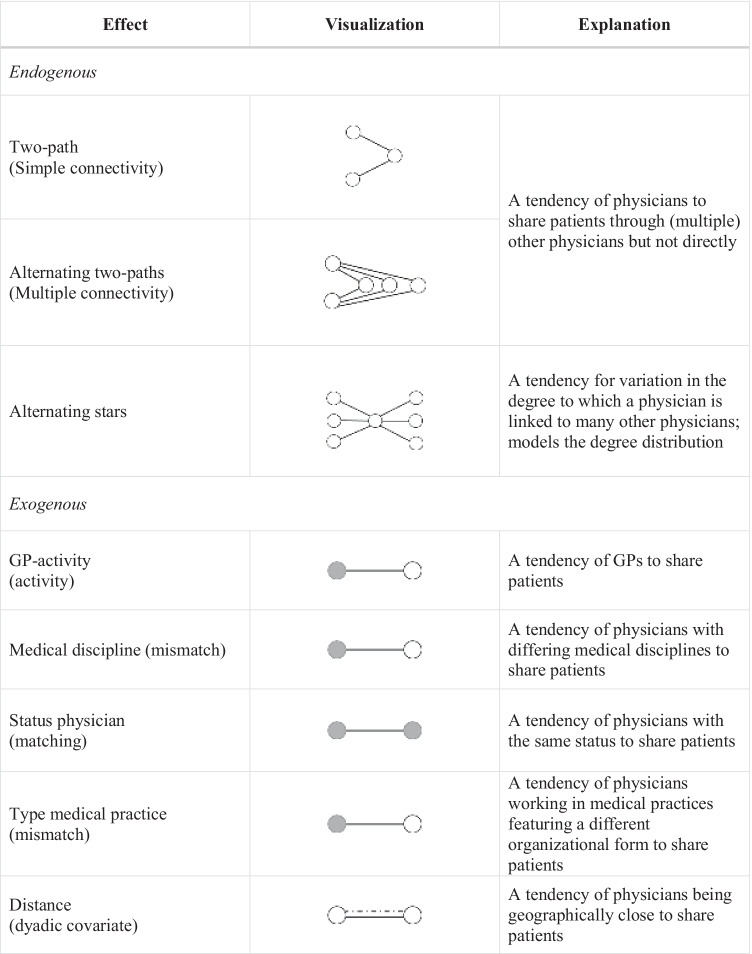
Further endogenous and exogenous effects

GP = general practitioner (with pediatricians)

We used maximum likelihood estimation to explore which patterns affect the presence of patient-sharing ties in the two networks. For a further, more detailed explanation of the examination process, we refer to Lusher et al. [67]. Based on Robins and Lusher [68], we assessed the goodness of fit tests of the estimated ERGMs.

## Results

**Fig. 2:**
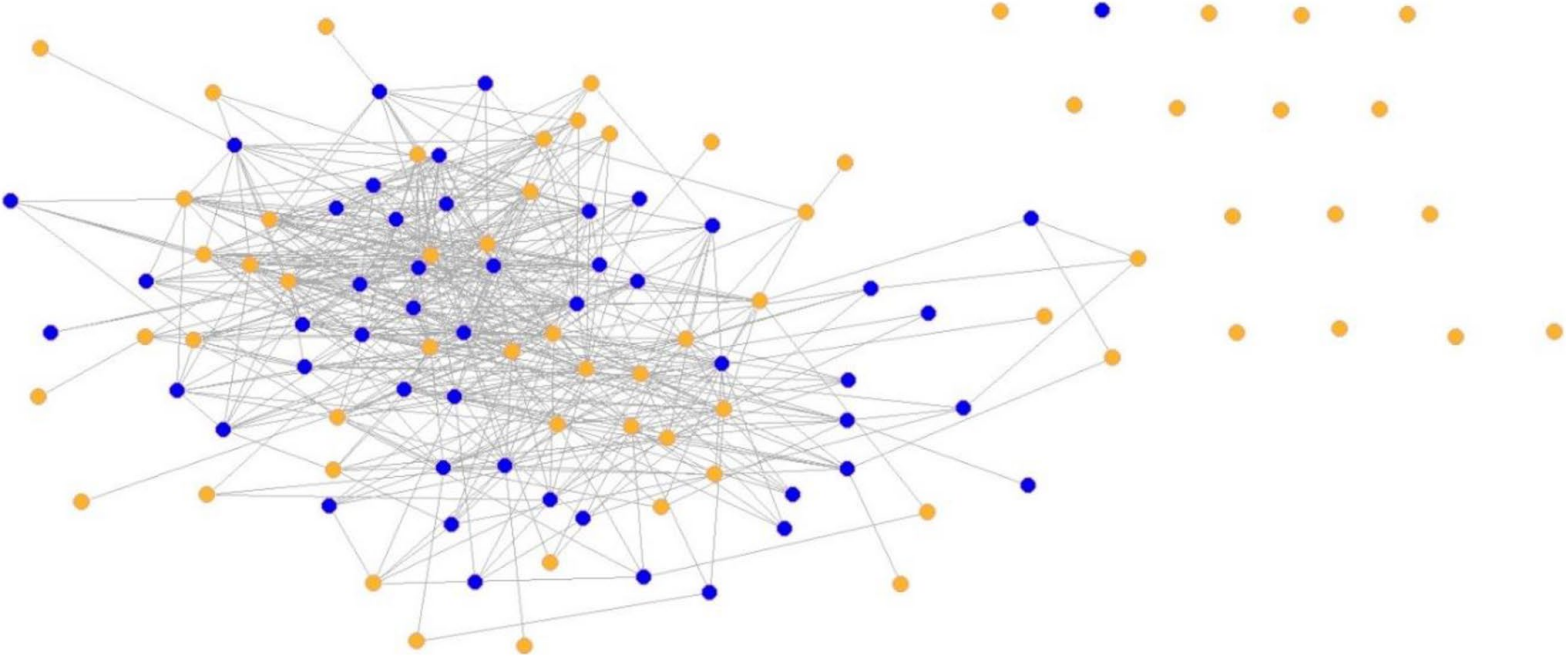
Visualization of the physician-induced network. *Notes*: Kamada-Kawai-Algorithm; blue nodes = general practitioner; orange nodes = medical specialist; 16 isolates

**Fig. 3:**
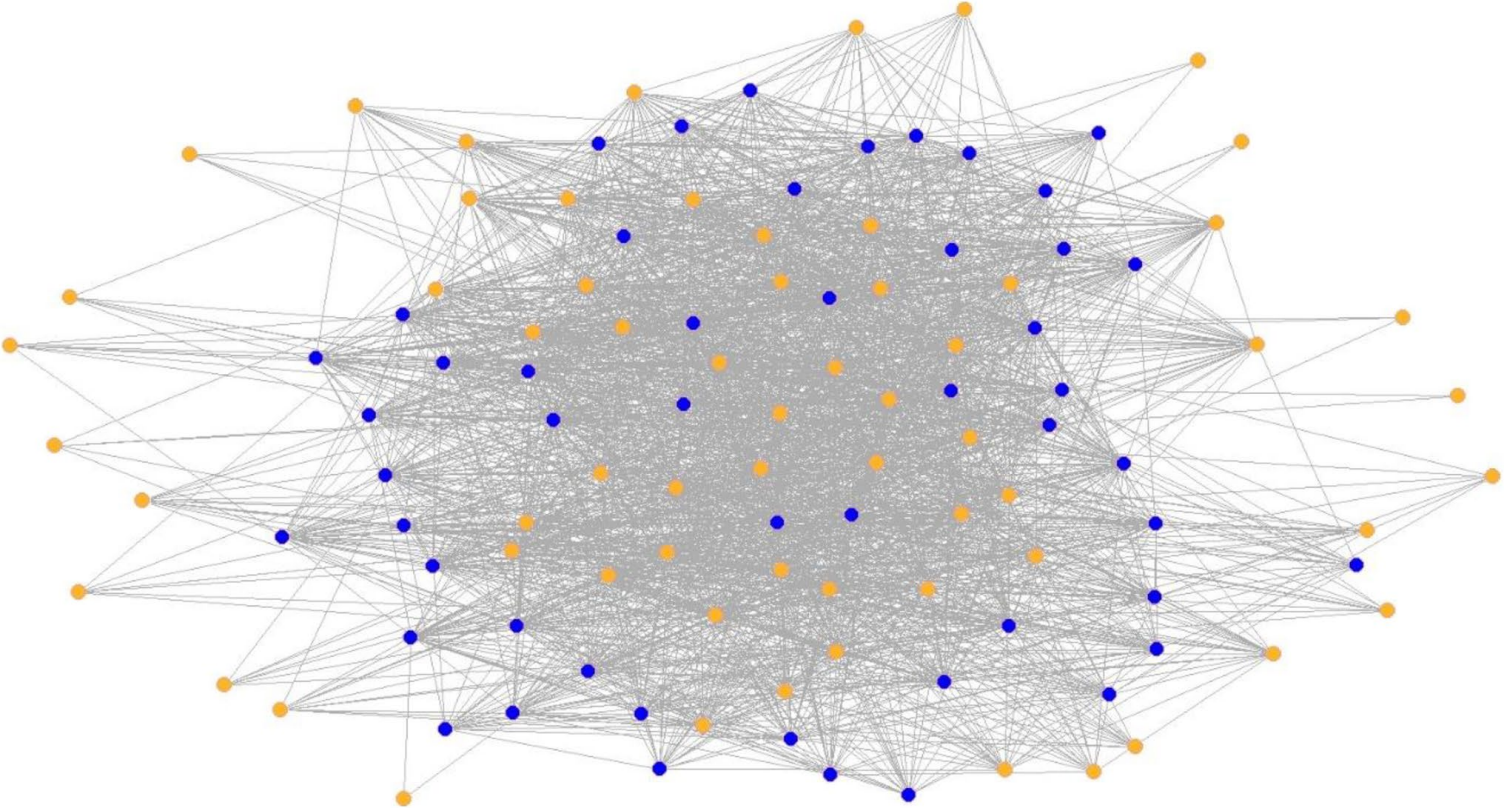
Visualization of the patient-induced network. *Notes*: Kamada-Kawai-Algorithm; blue nodes = general practitioner; orange nodes = medical specialist; no isolates

Before we interpret the results, we first describe the patient-sharing network and differentiate between physician- and patient-induced network structure. 113 physicians are involved in the provision of outpatient care for children and adolescents in the patient-sharing network. The network that only integrates the patient side has a higher density (39.6 percent) than the physician-induced network (8.7 percent). In the network initiated by patients, all office-based physicians are involved in sharing patients, whereas in the network arranged by the physicians through referrals, it seems that some physicians are not recommended, represented through isolated actors. In Figs. 2 and 3, the two networks are visualized. In the supplementary material, Table 1 summarizes the descriptive statistics for the physician- and patient-induced network [69].

Tables 3 and 4 present the results of the ERGMs for the physician- and patient-induced networks. Table 3 shows the results for the closed and open triadic network structures. Results for further endogenous and exogenous effects are presented in Table 4. The goodness of fit (GOF) tests suggest a satisfactory model fit [68, 70].^2^

**Table 3:**
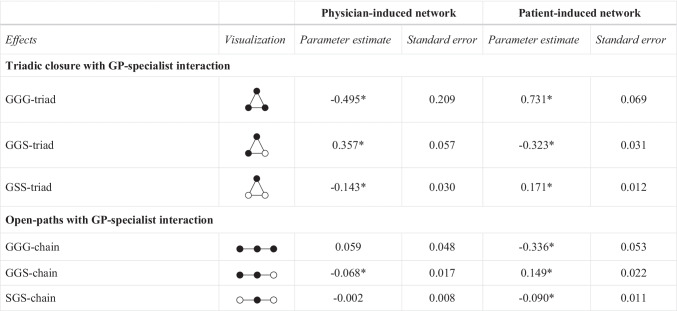
Results of the ERGMs

*Significant at p ≤ 0.05

G = general practitioner (with pediatricians); S = Medical specialist*.* λ = 2 if not indicated otherwise. Larger lambda values for structural parameters were selected to control for dense regions within the networks and to achieve convergence or to get a satisfactory model fit (for further details see [68]). Density were fixed to assist convergence [71]

**Table 4:**
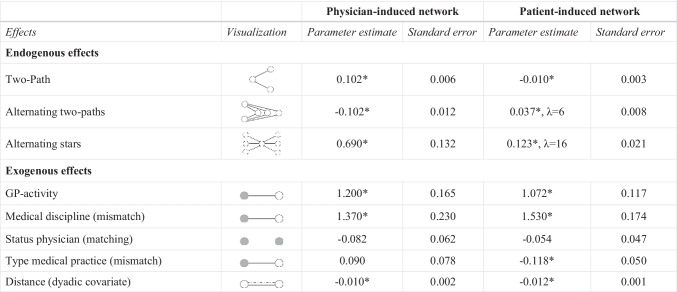
Results of the ERGMs—Further endogenous and exogenous effects

*Significant at p ≤ 0.05

GP = general practitioner (with pediatricians)*.* λ = 2 if not indicated otherwise. Larger lambda values for structural parameters were selected to control for dense regions within the networks and to achieve convergence or to get a satisfactory model fit (for further details see [68]). Density were fixed to assist convergence [71]

In general, the results reveal that the two networks can be characterized by different closed and open triadic patterns between GPs and medical specialists. Regarding the closed triadic substructure that includes only GPs (*GGG-triad*), we obtain a negative parameter estimate for the physician-induced network and a positive estimate for the patient-induced one. We expected this result in the first case. However, we did not anticipate a positive *GGG-triad* effect in the network initiated by patients, a result which indicates that patients get medical treatment via GPs building small groups or cliques consisting of three different GPs. Furthermore, results of the *GGS-triad* effect are in line with our expectations. There is a tendency for two GPs and one specialist to form closed triads if patient-sharing is initiated by physicians (positive *GGS-triad* effect). In contrast, *GGS-triad* formation is less likely in patient-induced networks (negative *GGS-triad* effect). Considering the triadic interaction among one GP and two specialists (*GSS-triad*), we obtain a negative parameter estimate for physician-induced sharing of patients and a positive estimate for the other network. The first result of this effect contrast with our reasoning based on institutional specifications.

In regard to the open triadic or chain-like substructures between GPs and specialists, we obtain results that differ in some respects from the considerations described above. The *GGG-chain* effect is negative in the patient-induced network, meaning that open triad formation involving only GPs is less likely. The same effect is insignificant in the network arranged by physicians. Moreover, a negative estimate for the *GGS-chain* effect in the physician-induced network implies that GPs do not link other GPs with medical specialists. In contrast, this open triadic mechanism occurs in networks that are rather initiated by patients (positive *GGS-chain* effect). We integrated a third open triad effect (*SGS-chain*) capturing the gatekeeping-principle according to which GPs take on the role of gatekeepers and link specialists. The results show that this effect is less likely to occur in patient-induced networks (negative *SGS-chain* effect). Concerning the physician-induced network, the *SGS-chain* effect is non-significant.

The structure of both networks can additionally be explained through purely structural and actor-related mechanisms. Regarding the physician-induced network, the positive *two-path* effect indicates that some physicians tend to act as gatekeepers, binding two unconnected physicians through the sharing of patients *(simple connectivity).* Accordingly, there is a general tendency towards the formation of chain-like structures. Taking into account certain combinations of GPs and specialists, however, it is shown that some chain-like structures are unlikely to occur. In addition, there is a tendency against *multiple connectivity* (negative *alternating two-paths* effect). Thus, physicians do not generally share patients indirectly through multiple other physicians. The two parameters corresponding to *simple* and *multiple connectivity* yield opposite results with respect to the patient-arranged network (negative *two-path* effect; positive *alternating two-paths* effect). Physicians are connected through multiple others if the presence of patient-sharing ties is traced back to patients’ activities. The *alternating stars* effect was integrated to model the degree distribution. In both networks, we obtain positive parameter estimates indicating that degrees are not evenly spread among physicians which points towards centralized patient-sharing networks [66, 70].

We obtain significant results for almost all exogenous effects. Only the homophily effects for physicians’ status are insignificant in our models. The positive *GP-activity* effects indicate that GPs tend to form patient-sharing relations, regardless of whether the network was initiated by the physician or the patient. This suggests that the GP is frequently the first point of contact and coordinates the course of treatment for many patients. Furthermore, physicians belonging to dissimilar medical disciplines are more likely to share patients in both networks. This effect also considers exchange processes between different specialists (*medical discipline* mismatch effect). However, shared treatment rather occurs among physicians working in practices with the same organizational form. While the parameter estimate for the practice type is not relevant in the physician-induced network (insignificant *mismatch* effect), sharing patients across dissimilar types of practices in patient-induced networks occurs less often than expected by chance (negative *mismatch* effect). The two negative distance effects confirm that spatial proximity positively affect the formation of patient-sharing ties which is in line with previous results [e.g. 22, 32].

## Discussion, conclusion, and future research

We take a comparative approach and investigate the structure of patient-sharing networks initiated by physicians and those that originate from patient behavior. By using the referral information available in the medical claims database, we derive two divergent networks based on shared patients. Based on certain institutional expectations, which mainly apply in healthcare systems following the Bismarck model, we study different triadic tendencies. We focus on open and closed triadic patterns between GPs and various medical specialists, applying a class of ERGMs. To some extent, the results confirm that outpatient treatment complies with the institutional requirements that are imposed directly and indirectly on physicians and patients. However, some findings are inconsistent with the systemic guidelines, suggesting that other factors may influence the triadic exchange between office-based physicians. Our analysis reveals the “structural realities” that are present in both networks.

First, the sharing of patients preferably take place between complementary physicians. It seems that GPs respect the principle of service complementarity and do not form small groups with other GPs based on referrals. Similarly, patients should adhere to this principle by opting not to solicit second or third opinions, which would save expenses and resources on the supplier side, for example [43]. The empirical results lead to the conclusion that patients do not necessarily comply with this guideline. Closed triads between GPs occur in patient-induced networks. A possible explanation for this observation is that patients prefer to seek a range of medical opinions. By selecting children and adolescents as patients for our study, it is likely that in some cases second or third opinions will be requested. For instance, parents consult a number of GPs in the event that a diagnosis seems unclear or if the initial treatment was not satisfactory [47]. In addition, contractual arrangements [25, 45], such as the replacement of a physician who is on vacation may further clarify why patients link GPs together. If the permanent GP is absent, other GPs who are located nearby will often act as substitutes for this physician. The patient consults a partner of the permanent GP, a process which generally proceeds without a referral. Further formal and informal agreements between GPs also result in interactions based on common patients [72]. It is worth noting that pediatricians are defined as GPs in this study, which might also help to explain the triadic exchange exclusively between GPs.

Second, the expectation that GPs will act as gatekeepers, bringing complementary specialists together through referrals and thus leading to the formation of a closed triad in the physician-induced network, is not fulfilled. One reason for this may be that there are no referrals between certain specialists or that the GP links two physicians with the same speciality who do not work together. Contrary to the requirements that patients should ideally be referred to suitable specialists, it seems that patients consult a GP and two (complementary) specialists independently, thus creating closed triads involving these actors. The free choice of physician allows the patients full latitude to decide where to go for treatment [17]. As a result, patients visit physicians of the same speciality to ask for second opinions, thus tying similar experts to one another. Or patients may be treated directly by a medical specialist and bypass the gatekeeper principle altogether [33], which is desirable but not mandatory for patients in the German healthcare system, for instance [45]. In addition, some physicians forward patients to certain other physicians without a referral being made. Personal and informal relationships influence this procedure [46].

Third, our results indicate that open paths including two GPs and one specialist do not seem to determine the structure of physician-induced networks. Triads containing this combination of actors tend to be closed in networks arranged by office-based physicians, as physicians prefer to share patients with partners of their partners, thus forming small groups. One possible implication of this is that physicians make referrals to others also linked to each other, even if the physicians share the same institutional role. Knowledge gaps, mutual advice and increasing specialization in primary care are some possible reasons for referrals also taking place between GPs, especially when patients suffer from complex or rare diseases [15, 21]. In addition, non-medical reasons, such as joint memberships in organized networks or associations, contractual agreements, or mutual attendance in training courses, may positively influence the shared treatment of patients. Regular meetings can lead to social and trust-based partnerships between physicians, which possibly encourages the issuance of referrals among the physicians involved [73].

In contrast, if patients initiate the network, we obtain an inverse result. Multiple examinations by the same provider type should normally be avoided in order to limit unnecessary treatments and reduce capacity constraints [43]. Furthermore, the decision on whether to seek out specialist treatment should be made by the GP in the form of a referral. The patient-induced network is characterized by an open path consisting of two GPs and one specialist. This outcome is not in line with the institutional expectations. It could be the case that patients misuse their right to autonomously determine the course of treatment and consult an additional GP, who is not their regular physician, as well as a specialist. However, also in this case, the increasing specialization of primary care and the inclusion of pediatricians as GPs could also play a role in explaining patient-sharing among this group of actors. In addition, the patient-induced network is not designated by an open triad, in which a GP links two specialists. In connection with the positive result for the closed triadic structure involving the same combination of actors, it can be stated that patients rather consult a GP and medical specialists that are connected to one another. In the supplementary material, Table 2 summarizes the findings of the triadic structures for the physician- and patient-induced network [74].

We take a closer look at patient-sharing networks and differentiate between connections that are more likely to result from physician activities and those based more on patient behavior. Previous studies defined the sharing of patients as a collaborative relationship among physicians or other types of healthcare providers [75, e.g. 76]. They presuppose that the providers of healthcare services are mainly responsible for the formation of patient-sharing ties. This assumption is valid for some healthcare networks. In many outpatient care settings, the exchange is rather initiated by patients. Our empirical study confirms that this complex type of interaction should be examined more closely. The structures of physician- and patient-induced networks differ with regard to the outpatient environment. Beyond that, by adopting a triadic view, we identify variations in the interaction between GPs and medical specialists. Going beyond the dyadic level enables us to detect how office-based physicians cluster when the principle of service complementarity is included in triadic exchange. Researching various triad types reveals that physicians and patients do not necessarily act as institutionally intended. The results highlight the “structural realities” of both networks. Our study contributes to research examining whether institutional expectations are fulfilled in networks arranged either by physicians or patients by considering various exchange possibilities among GPs and specialists in treating common patients.

These insights are of great importance for policymakers and healthcare administrators, as it allows them to monitor the extent to which physicians and patients follow predefined rules or desired practices from an institutional perspective. Overall, our findings suggest that patients tend to decide for themselves which physicians to consult and referral and thus coordination by physicians seems to be the exception. What is more, patients do not necessarily follow the principle of service complementarity but connect physicians that occupy the same institutional role. This leads to the consideration of whether and to what extent the degree of freedom of the patient should be restricted in the future to avoid malpractice. The identification and comparison of specific triadic structures in referral networks and in networks based on patient self-organization is therefore relevant for decision-making processes at the macro level [47]. In addition, the exploration of the patient-induced network reveals “hidden” treatment paths not perceived by the physician. This could be used to locate existing networks and draw physicians’ attention to them [44]. The network-based investigation of existing links can be applied as a supportive tool for managing treatment processes and the utilization of outpatient medical services. The implementation of organized treatment processes and new (integrated) care models, e.g. GP-centered concepts in the German outpatient system [45, 48], can thus be facilitated with this knowledge.

There are some limitations in our investigation that should be addressed in future studies. To deduce physician- and patient-induced networks, we used the referral information available in the medical claims data. We defined patient-sharing ties that came about due to referrals as physician-induced linkages [33]. Links without any referral details were allocated to the patient side. But patients can actively influence the referral process. Some insist on further treatment, which is usually provided by specialized professionals. Given our use of medical claims data, it was not feasible to isolate the patient’s involvement from the referral activity. Conversely, physicians can advise the patient to consult a specific physician without providing a referral. This means that the physician can also shape the patient-induced network. Further research, perhaps of qualitative nature is required to separate the two networks even more precisely. It was not possible to infer the direction of the patient-sharing relations from the data. The temporal course of patient treatments was not accessible. To shed more light on the triadic exchange and to be able to make statements about the sequence of patient treatment (for instance, a GP sends patients to two specialists but only receives patients from one), directed patient transfer networks need to be examined. In addition, we were unable to deduce the course of treatment of a single patient. It would be interesting to analyze individual treatment pathways to find out the extent to which the same patient is contributing to the closure of a triad in the patient-induced network. Thereby, it might be possible to more accurately determine whether the patient adheres to institutionally intended treatment procedures. Furthermore, the results of our study are based on a regional patient-sharing network in Germany. The triadic mechanisms associated with the institutional role of physicians should be explored in other districts, and additionally in further countries, with the aim of drawing more general conclusions. Even though healthcare systems differ in terms of their institutional frameworks and regulatory principles, our empirical study proposes one approach for comparing cooperation and exchange patterns within and across healthcare systems.

## Supplementary Information

Below is the link to the electronic supplementary material.

Supplementary file1 (PDF 226 KB)

## Data Availability

We would like to thank the National Association of Statutory Health Insurance Physicians (Germany) for providing the data. An approval of an ethics committee was not necessary.

## Footnotes

1 In the German healthcare sector, the so-called requirements planning governs local outpatient care. The aim is to guarantee needs-based and consistent healthcare services. The spatial planning scheme used in the requirements planning, is based on the Bundesinstitut für Bau-, Stadt- und Raumforschung (BBSR) [52]. The district boundaries date from 2011.

2 Patient-induced network: Single t-values for network configurations not included in the ERGM (t-values of four extra-dyadic effects for distance), range between 3.00 and 4.96, which is not in line with the criteria suggested by for instance Robins et al. [70]. These inadequacies have already been noted in previous studies [e.g. 77]. Further model specification is needed to obtain adequate values for the extra-dyadic effects of the patient-induced network.

## Code availability (software application or custom code)

We used the PNet software for analyzing the data.

Peng Wang, Garry Robins, Philippa Pattison, Johan Koskinen (2009) PNet: program for the simulation and estimation of exponential random graph models. Melbourne School of Psychological Sciences, The University of Melbourne.
