# Supplementary material for: Beyond patient-sharing: Comparing physician- and patient-induced networks
Source: Health Care Manag Sci. 2022 Jun 1;25(3):498–514. doi: 10.1007/s10729-022-09595-3 (PMC9474566; doi:10.1007/s10729-022-09595-3)
Supplement: Supplementary file 1 — Supplementary file1 (PDF 226 KB) [file 10729_2022_9595_MOESM1_ESM.pdf]

Supplementary Material

Table 1: Descriptive statistics for physician- and patient-induced network

	**Physician-induced network**	**Patient-induced network**
**Network characteristics**		
Number of actors	113	113
Density	0.087	0.396
Number of ties	1106	5022
Average degree	9.788	44.442
Number of isolates	16	0
Number of patients<br>(children and adolescents; aged 0-17 years)	1620	7448
**Actor-based characteristics**	**Both networks**	
Institutional role	(1) GPs (with pediatricians included): 44.2 %<br>(0) Medical specialists: 55.8 %	
Medical disciplines	Range: 1 - 18 different medical disciplines	
Status physician	Licensed physicians: 77 %<br>Employed physicians: 18.6 %<br>Authorized physicians and other status: 12.5 %	
Type of medical practice	Single practice: 58 %<br>Group practice: 23 %<br>Ambulatory healthcare center: 14.2 %<br>Authorized ambulance near hospital, emergency practice and other: 7.2 %	
Distance (travel time in minutes)	Mean: 49.25; SD: 20.92<br>Max.: 100.29<br>Min.: 0	

Note. GP = general practitioner

Table 2: Findings of triadic structures for physician- and patient-induced network

**Effect name**	**Visualization**	**Physician-induced structure Network with referral information**		**Patient-induced structure Network without referral information**	
		Expected result	*Empirical support*	*Expected result*	*Empirical support*
GGG-triad	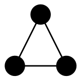	Unlikely to occur	✓	Unlikely to occur	✗, positive effect
GGS-triad	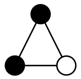	Likely to occur	✓	Unlikely to occur	✓
GSS-triad	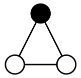	Likely to occur	✗, negative effect	Likely to occur	✓
GGG-chain	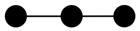	Unlikely to occur	✗, non-significant	Unlikely to occur	✓
GGS-chain	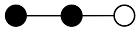	Likely to occur	✗, negative effect	Unlikely to occur	✗, positive effect
SGS-chain	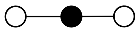	Likely to occur	✗, non-significant	Likely to occur	✗, negative effect

● = general practitioner with pediatricians (G/GP); ○ = medical specialist (S/M)
